# Institutional Questions

**Published:** 1900-08-04

**Authors:** 


### INSTITUTIONAL QUESTIONS.

### Military and Naval Hospitals.

A correspondent writes: I am anxious to obtain all the practical information I can get as to Red Cross Societies, and especially respecting the Hospital Ships in South Africa, as regards cost, proficiency, and a due return for money expended on them. The point arises as to whether a base hospital or sanatorium might not be more efficient and serve a larger number of sick and wounded. I should like to have any English or American publication dealing with these points.

*** There is no special information of a reliable or exhaustive character concerning the hospital ships in South Africa on the points you mention at present. That information will no doubt be forthcoming when there has been sufficient time to test their utility in practice. As it is, they have in practice only just settled down to work. "Hospitals and Asylums of the World" (London: The Scientific Press), Volume III., contains a long chapter (No. 32) on military and naval hospitals throughout the world, and also much information on the subjects you refer to.

